# Identifying Interaction Patterns of Tangible Co-Adaptations in Human-Robot Team Behaviors

**DOI:** 10.3389/fpsyg.2021.645545

**Published:** 2021-07-19

**Authors:** Emma M. van Zoelen, Karel van den Bosch, Matthias Rauterberg, Emilia Barakova, Mark Neerincx

**Affiliations:** ^1^Interactive Intelligence, Intelligent Systems Department, Delft University of Technology, Delft, Netherlands; ^2^Human-Machine Teaming, Netherlands Organization for Applied Scientific Research (TNO), Soesterberg, Netherlands; ^3^Department of Industrial Design, Eindhoven University of Technology, Eindhoven, Netherlands

**Keywords:** human-robot collaboration, human-robot team, co-adaptation, embodiment, interaction patterns, emergent interactions

## Abstract

As robots become more ubiquitous, they will increasingly need to behave as our team partners and smoothly adapt to the (adaptive) human team behaviors to establish successful patterns of collaboration over time. A substantial amount of adaptations present themselves through subtle and unconscious interactions, which are difficult to observe. Our research aims to bring about *awareness of co-adaptation* that enables team learning. This paper presents an experimental paradigm that uses a physical human-robot collaborative task environment to explore emergent human-robot co-adaptions and derive the interaction patterns (i.e., the targeted awareness of co-adaptation). The paradigm provides a tangible human-robot interaction (i.e., a leash) that facilitates the expression of unconscious adaptations, such as “leading” (e.g., pulling the leash) and “following” (e.g., letting go of the leash) in a search-and-navigation task. The task was executed by 18 participants, after which we systematically annotated videos of their behavior. We discovered that their interactions could be described by four types of adaptive interactions: stable situations, sudden adaptations, gradual adaptations and active negotiations. From these types of interactions we have created a language of interaction patterns that can be used to describe tacit co-adaptation in human-robot collaborative contexts. This language can be used to enable communication between collaborating humans and robots in future studies, to let them share what they learned and support them in becoming aware of their implicit adaptations.

## Introduction

With AI being increasingly used in social robotics (Breazeal et al., 2016), there is a growing number of possible applications in which artificially intelligent robots need to interact and collaborate with humans in the physical space. Creating AI for the physical world comes with many challenges, one of which is ensuring that a robot does not only execute its own task, but instead behaves as a team partner, to enable human and robot to become one well-functioning unit of collaboration. One of the mechanisms that can be used to enable this, is a process of co-adaptation, where both human and robot, through (physical) interaction, adapt their behavior to develop successful patterns of collaboration over time (Chauncey et al., 2017).

To define what we mean by co-adaptation, we can think of how humans adapt their behavior in a reciprocal manner when they collaborate with other humans: the kind of adaptive interactions they use to achieve a fruitful collaboration. It is known that a human team’s ability to adapt to new circumstances is vital for its performance, and team members tend to rapidly develop updated interaction patterns that fit with new situations (Burke et al., 2006; Uitdewilligen et al., 2013). Humans have the ability to intuitively interpret body language of their team members and to send signals when initiating adaptations (Sacheli et al., 2013). This kind of non-verbal interaction is not obvious when a team member is a robot. While we migh1t be able to interact with a robot using language, collaborative interactions are generally multimodal and contain many subtle and implicit non-verbal interaction cues that help us to create tacit knowledge. The focus of this paper is on these non-verbal interactions, and specifically those that are connected to physical contact.

Two classic examples of non-verbal interactions for co-adaptation in a human-non-human collaborative context can be found in human-animal interaction:

(1) The interaction between a horse and its rider (Flemisch et al., 2008);
(2) The interaction between a guide dog and a blind person (Lagerstedt and Thill, 2020).

When a human rides a horse, they start off as two separate entities with their own goals. As they interact for a longer period of time, they gradually start to better understand the other, adapting their interaction concurrently, until they become one joint system acting toward a common goal through subtle and implicit interactions. Another example is the interaction between blind people and their guide dogs: blind people truly need to trust and follow the choices of the guide dog, whereas in horse riding, the human makes most of the decisions. When guide dogs and blind people learn to navigate together, the human needs to learn to assess when to adapt its behavior to follow the dog, and when to give the dog directions about their route. The dog must learn to understand what the human is and isn’t comfortable with and adapt its behavior to that. All this learning and adapting takes place through subtle physical interactions.

Mechanisms of adaptation have been studied in intelligent agents, more specifically in the field of multi-agent systems [e.g. (Foerster et al., 2016; Iqbal and Sha, 2019)]. Research addresses learning algorithms, such as different types of Reinforcement Learning, and investigates their effects on agent performance or team performance. Little to no attention is paid to the interactions that the agents engage in, which bring about the adaptations [except for some examples such as (Baker et al., 2020)]. Even when mechanisms of adaptation are studied in human-robot interaction contexts such as in Nikolaidis et al. (2017a), the effects on performance are studied. We believe that research should also address the interactions that bring about successful adaptation, to come closer to the fluency and naturalness of the above-mentioned human-animal examples.

There is a need for further study of the specific interactions and interaction patterns that bring about co-adaptation when humans and robots collaborate. A deeper insight in these interactions and patterns can help researchers and designers to study and create more natural and fluent human-robot collaborations that take the limitations and affordances of the physical world into account. In addition, such insights can support the collaborating human and robot to become more aware of their implicit adaptations and communicate about them, to further improve their collaboration. In Section “Co-Adaptation in Human-Robot Teams,” we define co-adaptation in a human-robot collaboration context, and we explain the relevance of embodiment in this process in Section “Research Challenge.” We describe an experimental paradigm that we designed and implemented to conduct an empirical study into co-adaptation and how it emerges from interactions. This human-robot team task was presented to human participants, after which we analyzed the team behavior in terms of interactions and interaction patterns. The resulting interaction pattern vocabulary and language provides a thorough analysis of co-adaptive interactions surrounding leadership roles in human-robot teams.

## Co-Adaptation in Human-Robot Teams

### Co-Adaptation - A Definition

In human-only teams, the term ‘team adaptation’ is used to describe the changes that occur in team behavior and performance. More specifically, (Burke et al., 2006) define team adaptation as “a change in team performance, in response to a salient cue or cue stream, that leads to a functional outcome for the entire team”. They describe that it “is manifested in the innovation of new or modification of existing structures, capacities, and/or behavioral or cognitive goal-directed actions” (p. 1190). On top of that, it is argued that an important aspect in this is that the team members update their mental models according to changes in the task situation (Uitdewilligen et al., 2013).

We use the term *co-adaptation* instead of team adaptation, as we study the adaptive interactions at the level of the individual actors: team adaptation is a result of adaptative behavior exhibited by the individual team members. Also, co-adaptation is used more often in the context of (physical) human-robot interaction. We define co-adaptation in human-robot teams as follows:

*A process in which at least two parties change their behavior and/or mental models concurrently as a consequence to changes in task or team situation while collaborating with each other.*

This concurrent changing of behavior and/or mental models is relevant for the team, as smooth collaboration requires partners to adapt to each other over time. Since humans are adaptive creatures by nature, and artificially intelligent systems are becoming more and more adaptive, there is an opportunity to study how they adapt together as they collaborate.

Co-adaptation is a process which generally takes place over a short period of time, e.g., over the course of several seconds or minutes; this timespan is generally considered in the study of co-adaptive behaviors (e.g., in Nikolaidis et al. (2017a), see also Section “Related Work”). It is not necessarily a deliberate process: adaptation happens as a consequence of interactions and an implicit or explicit drive to improve performance or experience. The resulting behaviors or mental models in both adapting partners do not necessarily persist over time and contexts, as new contexts and influences may cause the co-adaptation to continue. We used the above definition to describe co-adaptation as a design pattern in Table 1 [according to the template specified in van Diggelen et al. (2019)]. This table provides a detailed explanation of the possible positive and negative effects of co-adaptation, as well as an overview of the kind of contexts in which it is relevant to develop or apply co-adaptation.

**TABLE 1:** Proposed Design Pattern for Co-adaptation.

**Proposed Design Pattern for Co-Adaptation**	
Behavior patterns	Team members engage in collaboratively solving a task. While they do this, they observe each other’s actions and adapt their behavior in an attempt to make the collaboration more fluent and effective.
Potential positive effect	The performance on the collaborative task increases. Both partners will be able to work more efficiently, as there is less idle time, fewer mistakes and more understanding between the partners
Potential negative effect	In the process of adapting, there is a risk of making mistakes. In addition, it takes time to adapt to a working strategy, which might have negative effects on the immediate performance.
Use when	Team partners need to collaborate but don’t know the best strategy to complete the task. At the same time, the task and capabilities of the team members contain many implicit aspects that are hard to explicitly communicate or make agreements about.
Example	A human and a robot arm have to collaboratively assemble a product. There are different parts that either of them can assemble, and some parts need to be jointly assembled; e.g., the robot needs to hold up a heavy part while the human adjusts the bottom. If the human has to constantly provide the robot with instructions, this will slow them down, so it is useful to let the robot move autonomously and to synchronize their actions. When they start collaborating, the human might not trust the robot enough to adjust the bottom of a part that the robot holds up, in fear of being crushed underneath the part. The robot might see the hesitation and move the part upside down, such that the human can reach the object more easily. In turn, the human will have to adjust their workflow to do their task, but the fact that the robot adapted might increase the trust and understanding between the partners, which can in turn improve future team performance. While adapting, however, the human might make the mistake of trusting the robot too much, and think they can climb on top of the heavy part whereas the robot is unable to hold that weight. The co-adaptive process, if done too quickly or inconsiderate, therefore has the risk of making mistakes that hamper immediate performance.
Design rationale	A process of mutual adaptation helps to establish and maintain common ground, one of the main aspects of necessary for enabling collaboration between humans and machines (Klein et al., 2004; Sciutti et al., 2018). This might also be called mutual understanding, meaning that both parties are able to predict and/or explain each other’s actions, leading to trust and eventually smooth collaboration (Azevedo et al., 2017). In human-only teams, co-adaptation leads to team adaptation (Burke et al., 2006), which has shown to be an essential aspect of successful teams.
Type	Collective

### Related Work

In the sections below, we discuss related work on co-adaptation in human-robot or human-AI collaborative contexts, as we are studying interactions that bring about co-adaptation. Since we are specifically interested in analyzing and categorizing interactions and interaction patterns, we also looked at literature on interaction taxonomies within collaborative contexts. There is a body of research on dynamic role switching in human-robot collaboration, which has many similarities with how we described co-adaptation in terms of interactions. However, the existing literature [e.g. (Evrard and Kheddar, 2009; Mörtl et al., 2012; Li et al., 2015)] focuses on computational approaches to enable a robot to dynamically switch roles in an attempt to optimize performance of a human-robot team. While the existing studies evaluate the impact of the robot strategies on human factors, they do not study the natural interactions between the human and robot that arise as a consequence of the necessity for role switching. Therefore, we do not go into further depth on these papers.

#### Co-Adaptation

Most work on human-agent co-adaptation focuses on making the agent adaptive to the human, using information on different properties of the human [e.g. (Yamada and Yamaguchi, 2002; Buschmeier and Kopp, 2013; Gao et al., 2017; Ehrlich and Cheng, 2018)]. There have been studies that investigated how a human adapts in situations when collaborating with an intelligent agent, using the team’s performance to determine the impact of co-adaptive collaborations (e.g. (Mohammad and Nishida, 2008; Youssef et al., 2014; Nikolaidis et al., 2017a, b). In addition to determining the effects of co-adaptation on performance, it is also necessary to study the kind of interactions that emerge throughout the co-adaptation process and support team members in the process of developing a fluent collaboration. A better understanding of these processes will help to initiate and maintain co-adaptation in human-agent teams.

(Xu et al., 2012) have outlined requirements for co-adaptation to occur in human-robot teams. First, they argue that in order to achieve a common purpose, both agents need to be prepared to adapt their behavior to their partner, should actively and dynamically estimate the partner’s intention, and develop options of how to adapt their own behavior in response. Another requirement is that the agents need to be able to receive and appreciate feedback or reward from the other, to express their internal state to their partner in a comprehensible manner, and to establish with their partner a common protocol for interaction. Third, the authors outline several inducing conditions, derived from experimental work, that can be used to ensure a mutually adaptive process will start, for example that both agents should be able to take initiative.

We formulated our own requirements for a task environment that would fit with our research goals of studying co-adaptive interactions, which include the mixed-initiative requirement as well as the requirements for dynamic and adaptive behavior (which we connected to an improvement in team performance). Moreover, we added two requirements that relate to the presence of a common ground (general common ground as well as interaction symmetry). Common ground is considered to be necessary for any collaboration (Klein et al., 2004), while interaction symmetry is often used to provide the possibility for imitation, which can create initial common ground [e.g. in Sasagawa et al. (2020)]. A full description of these requirements is given in Table 2.

**TABLE 2:** Task requirements for a collaborative, co-adaptive task environment.

**Task requirements**	
Mixed Initiative	Both parties can take the initiative for an interaction at any point in time [see (Xu et al., 2012)]
Interaction symmetry	Interaction modalities should have a certain level of symmetry, meaning that there is at least some overlap in the way the two parties can interact with the other, to enable imitation. Interaction symmetry thereby contributes to the common ground.
Performance improvement	By adapting their individual behavior, team members can support an improvement in team performance.
Collaborative advantage	It must be easier to be successful at the task when collaborating, as opposed to doing it on your own
Common ground	There must be a common ground between the collaborating partners. In our case this comes from the physical nature of the task

#### Interaction Taxonomies

The literature reports two important existing studies into interaction taxonomies that describe interactions in collaborative tasks. One of those papers describes a top-down approach of describing different types of interactive behaviors based on game theory (Jarrassé et al., 2012); the other describes a bottom-up approach where interaction behaviors were identified from empirical observations (Madan et al., 2015). Both taxonomies were validated on their applicability by successfully classifying behaviors in different HRI scenarios. Although useful to describe collaborative behavior, the top-down approach [as used in Jarrassé et al. (2012)] resulted in a taxonomy that describes interaction at a high level of abstraction (distinguishing for example between competitive versus collaborative behavior). Such a taxonomy can be used to describe the overall behavior in a task, but it does not provide insights into (atomic) interactions that drive adaptation. The taxonomy presented in the other paper (Madan et al., 2015) presents interactions at various levels of detail, where the highest level of detail describes categories of interactions (i.e., *harmonious*, *conflicting* or *neutral*). The lower level interactions are more closely related to what we are interested in. They describe interaction patterns such as *harmonious translation*, *persistent conflict* and *passive agreement*. These interaction patterns focus on interactions related to collaborative object manipulation and were observed in a specific controlled task environment. This leaves room to study interactions in other contexts, to investigate a wider range of possible interaction patterns. Moreover, they do not provide information on how the different interaction patterns relate to each other; how they follow each other or how one pattern leads to a specific other pattern. We believe that the relations between interaction patterns are especially important when looking at adaptation. In our study, we take a bottom-up approach to identifying interaction patterns, which is similar to the work of Madan et al. (2015). This means that we do not predefine or design interactions, but that we set up a task that allows participants to behave as naturally as possible, and treat the data collection and analysis as an ethnographic study. Since such an approach requires us to have as little assumptions about behavior which will be observed as possible, we do not use the existing taxonomies when identifying interaction patterns. In our analysis, we focus specifically on adaptive interactions, as well as on how the different observed behaviors relate to each other. We will reflect on how our findings overlap or differ to the existing work in Section “Relation to Existing Interaction Taxonomies,” to understand how they might complement or complete each other.

## Research Challenge

The goal of this study is to empirically investigate the interactions between humans and robots that underly their co-adaptation when jointly performing a task. A challenge is that the adaptive intentions and outcomes of interactions are often not directly clear and observable. Partners may themselves not be aware that their behavior is an adaptation to the developments, and may be a response to subtle cues, possibly processed unconsciously. In order to nevertheless investigate how such important processes take place in a human-robot team, the approach of observing and analyzing embodied human-robot team behavior was taken. Expressivity and intentionality of behavior plays a large role in embodied interaction (Herrera and Barakova, 2020). It is believed that in such a setting the subtle and perhaps unconsciously executed adaptations will be expressed by means of physical, embodied interactions, hence being accessible for observation and analysis.

The literature on using embodied intelligence when studying human-robot interaction shows two main lines of research:

• A line of research that focuses on human cognition: investigating how computers or robotic interfaces can be used to understand and extend human cognition and behavior. An example of this is extending human cognition through prostheses or sensory substitution [e.g., as in Kaczmarek et al. (1991); Bach-y-Rita and Kercel (2003); Nagel et al. (2005)].
• A line of research that focuses on using embodiment to create more intelligent computers (or machines or robots). The robot’s intelligence is ‘grounded’ by a body with which it can interact with its environment [for example as described in Duffy and Joue (2000); Kiela et al. (2016)].

Our research approach is not directed at the intelligence of one particular partner of the team, but at the intelligence emerging from the interaction of partners. In the first described line of research (extending human cognition, e.g., using prosthetics), one of the main aims is to create a unity between the human and the added technology, such that for the human the artificial parts feels as though it is part of themselves. We research the unity of human and technology jointly forming a *team*, with both having a certain level of autonomy, and sharing a common goal. This approach distinguishes between cognition on an individual level (per agent) and collective cognition, at the team level. In our work, we focus on the team as a dyad formed by one human and one robot.

It is believed that studying embodied human-robot interaction, while they collaboratively perform a task, is an approach that can help us to discover and understand how a team adapts to the dynamics of the context, and how this adaptation emerges from the interactions between the team members.

## Materials and Methods

### Task Environment: Search-and-Navigation

We have developed a task in which a human and a robot jointly navigate through a space while searching for objects to collect additional points. The conditions and interdependencies described in Table 2 were implemented in this task.

**FIGURE 1:**
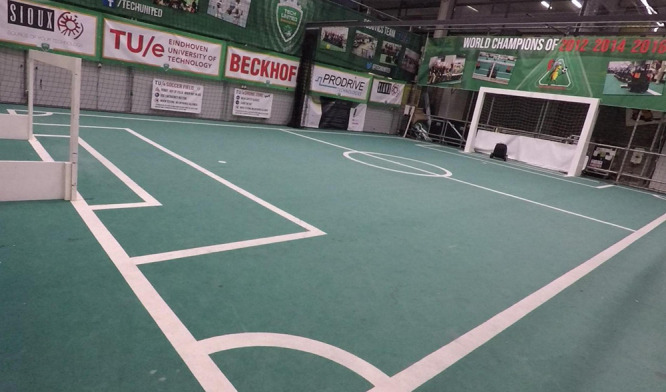
The field on which the task was executed. Participants moved from the goal on the left to the goal on the right (where the robot is stationed).

The team of human and robot are given the task of navigating between two points in space. The team’s assignment is to reach the goal location with as many points as possible. They start with 60 points, and lose a point each second until they reach the goal location. Virtual objects were hidden in the task area: some close to the shortest route to the goal location; others further to the side. Picking up a virtual object yields the team 10 points. These scores were chosen after trying out the task several times, such that solely focusing on the goal would yield approximately the same score as solely focusing on the objects, while combining the capabilities of both team members could potentially result in a higher score than either of the extreme strategies, ensuring a trade-off between the two. The partners have complementary capabilities: only the robot knows where the objects are; only the human can oversee the route and distance to the goal (see Figure 1 for an image of the field used). A sound cue is given when an object is picked up.

### Design of Human-Robot Interaction

**FIGURE 2:**
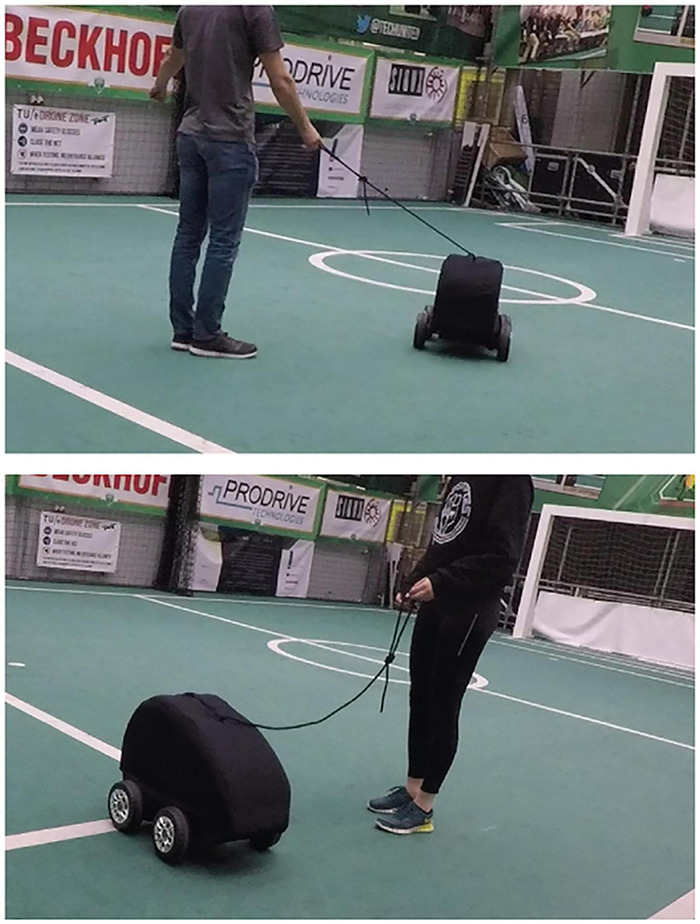
Two participants interaction with the robot showing a situation with a stretched leash and thus in a leading role (top) and situation with a loose leash and thus a following role (bottom).

We designed and implemented a remotely controlled robot with a leash (Figure 2). An ambiguous form was selected for the robot, without anthropomorphic features. This was chosen on purpose, to allow humans interacting with it to focus on the interaction, not on its form.

The leash was designed to be the only direct communication channel between the robot and a participant, to ensure specific evaluation of the interaction through the leash without too much noise of other interaction modalities. On top of that, the leash interaction allows for subtle and implicit interactions as both the participant and the robot can pull the leash more or less. The robot was explicitly made to be quite large and heavy, to allow it to pull the participant in a direction as well.

**FIGURE 3:**
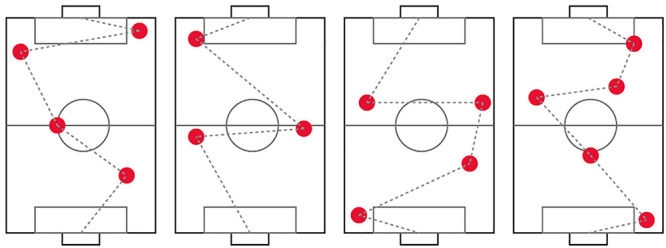
The four predefined maps with the locations of the objects (red circles), including a line indicating the default route of the robot. The bottom of the field is the starting point.

For our study, the robot was remotely controlled by a human operator (i.e. the experimenter). It is usually preferable that the operator is hidden from the participant, however, due to technical limitations this was not possible. The human operator was therefore on the field together with the participant and the robot during the experiment. A small pilot with two participants showed that participants only payed attention to the human operator in the first few seconds of the experiment, after which they directed their attention to the robot only. Therefore, and for practical reasons, we decided that it did not pose a problem for our study goals that the human operator was visible. The human operator controlled the robot according to a set of pre-defined rules: to direct the robot to the closest virtual object (following a default route as much as possible, as specified in Figure 3) if the leash was held loose by the participant (the operator, in contrast to the participant, knew the locations of all hidden virtual objects). If the participant kept a tight leash, the operator directed the robot to give in and to move toward the participant until the leash was no longer stretched. A detailed description of these rules is provided in Table 3. The human operator made decisions based on visual cues: they carefully watched the leash to see whether it was stretched. Human response time to visual cues is known to be on average 0.25 s, therefore, we can assume that the robot responded to participant behavior with a delay of 0.25 s.

**TABLE 3:** The protocol used by the human operator to control the behavior of the robot.

**Situation**	**Resulting Robot Behavior**
The leash is stretched	Follow the human in the direction that the leash is pulled in
The leash is loose AND the human-robot team is on the predefined route	Follow the predefined route
The leash is loose AND the human-robot team is not on the predefined route AND there are virtual objects that have not been picked up	Move toward the nearest virtual object that has not yet been picked up
The leash is loose AND the human-robot team is not on the predefined route AND all virtual objects have been picked up	Move toward the goal

The task and robot were designed such that both partners had their own knowledge, enabling them to initiate actions that their partner cannot initiate. The knowledge of both partners was relevant for the task, making collaboration beneficial and enabling the partners to learn how to use their knowledge in the best possible way. All communication and coordination between the human and the robot took place through the leash, which ensured that interactions are physically grounded, and allowed for subtle and implicit interactions.

### Experiment Setup and Initial Results

The experimental paradigm described above was previously used to study leadership shifts and its influence on subjective Collaboration Fluency in human-robot teams. This section will explain the experimental protocol used as well as results obtained in that study. For the current study, we have re-analyzed the data obtained in the original study to research specifically what interactions and interaction patterns bring about co-adaptation in such a task. In Section “Analyzing Behavior to Uncover Interaction Patterns” and “Data Analysis: Extracting Interaction Patterns” we will describe in detail how that analysis was done.

#### Experimental Protocol

Participants were told that they had to perform a collaborative task together with an intelligent robot, while holding the leash of the robot. They were presented with the described task and human-controlled robot, and were given instructions about how they could score points.

Before the start of the experiment, the participants were given the possibility to walk from one end of the field to the other with the robot. This was done to give participants an indication of the speed of the robot. After that, the first round started. The task was performed four rounds per participant. The locations of the virtual objects were different for every round. Four predefined maps with specified locations of the virtual objects were created for the human operator (Figure 3). Each of these maps were used for each participant during one of the rounds. The order of the maps was randomized for each participant to make sure that the observed behavior would not be influenced by the specific maps. After each round, participants were asked three interview questions:

(1) Can you explain the behavior of the robot?
(2) What was your strategy for completing the task?
(3) How did you experience the collaboration?

An overview of the answers given to these questions was given in van Zoelen et al. (2020). For the analyses described in the following sections, the answers to these interview questions were used to support the researchers in interpreting participant behavior.

#### Participants

A total of 18 people participated in the experiment (9 male, 9 female), consisting of students from different programs within Eindhoven University of Technology, with an average age of 23 (SD = 3.9). The participants were told that the person with the highest number of points on a single run would receive a gift voucher of €10 to motivate them to perform to the best of their abilities. Before the start of the experiment, participants gave their consent after carefully reading the consent form that explained all details of the experiment except for the focus of the research (evolving leadership shifts) and the specific behavior of the robot. After the experiment, they were debriefed on the exact purpose of the experiment.

#### Data Analysis: Coding Process

While performing the task, a camera placed in a corner of the field recorded the behavior of the participants. These videos were thoroughly analyzed through a process based on Grounded Theory (Charmaz, 2014), using different stages of open coding, closed coding, and categorizing. All videos were coded using an open coding process at first, to get a view on the different kinds of behavior present among participants as well as on events that triggered participants to switch between a more leading and a more following role. Using the results from the open coding, a coding scheme for closed coding was developed that contained codes describing task events, robot movement, participant movement, leash activity and the participant’s location relative to the robot. Each code was characterized as a leading, following or neutral behavior (see Table 4).

**TABLE 4:** The coding scheme that was developed to analyze the behavior of participants and the robot in the experiment.

**Code Category**	**Code**
Task events	Task is running
	Object sound
Robot movement	Standing still
	Moving toward object in goal direction
	Moving toward object away from goal
	Moving toward object across field
	Moving with participant
	Moving in goal direction
Participant movement	Standing still/waiting
	Moving around robot
	Moving in goal direction*
	Moving in robot direction
	Moving across field*
Leash activity	Loose
	Stretched*
	Pulled in direction*
	Loosening/stretching
Participant location relative to robot	Behind
	In front of*
	Next to

Codes marked with a * were considered leading behavior by the participant. The presence of these codes was taken as an indication for leading behavior in all further analyses.

All videos were then coded again using a closed coding process using The Observer XT (Noldus, 2019). This was done in an iterative manner, where each video was watched and coded again for each code category as specified in Table 4. Codes of different categories therefore could exist in parallel (e.g., codes for leash activity and codes for participant movement), while codes within a category (e.g., ‘loose’ and ‘stretched’) could not exist in parallel. An exception were the ‘task event’ codes; these were used to record how long it took participants to finish the task and to be able to see whether behavior lined up with task events. This left us with an overview of whether the participant was in a leading, following or neutral position across the three variables of leash activity, participant movement and participant location at each moment during the task. Combined with the visualization tool in The Observer XT, this enabled us to visually analyze the (development of) different behaviors across rounds simultaneously as well as to quantify the amount of leading behaviors present in each run. Intercoder reliability for the duration of sequences with another coder for 5.6% of the data (videos of 4 runs) was found to be 97.55%.

#### Previous Results

The task environment presented above has previously been described in van Zoelen et al. (2020). The main findings focused on three aspects:

• Interactions that trigger people to reconsider leadership roles;
• How leader/follower behavior changes over time;
• The interplay between subjective Collaboration Fluency and shifting leader/follower roles.

As the current paper builds upon and greatly extends the results presented in the previously published work, we will summarize these findings in the sections below.

#### Switch Triggers

An open coding process revealed six types of situations that typically triggered participants to reconsider whether they should behave in a more leading or following way. The first of those situations is at the start of the task, where participants express their initial idea about the role they should take on. The other five triggers are the following:

(1) Sound indicating a virtual object;
(2) A leash pull by the robot;
(3) The robot deviating from the route that leads to the final goal without clear leash pull;
(4) Getting close to the goal;
(5) The robot standing still.

Of these five triggers, numbers 1 and 2 are explicitly visible and clear moments in time, while numbers 3 and 4 are more implicit, slowly emerge and are harder to observe. Number 5 is a special case, as the robot standing still was sometimes clearly linked to the collection of a virtual object, but sometimes emerged more implicitly from the interactions in the task. Besides grouping them in explicit versus implicit triggers, they can also be grouped into task feedback (1 and 4) and partner feedback (2 and 3).

#### Leading Behavior Development

Apart from direct triggers for reconsidering leadership roles, we looked at how the level of leadership that participants expressed developed over the four different rounds in which the task was executed. Three different dimensions of behavior (leash activity, participant location relative to the robot, participant movement) were looked at separately. We found that for all these dimensions, six types of leadership behavior development could be observed, namely:

• Mostly following (a);
• Start off following, leading in the middle, following at the end (b);
• Start off following, increase of leading over time (c);
• Start off leading, increase of following over time (d);
• Start off leading, following in the middle, leading at the end (e);
• Mostly leading (f).

**FIGURE 4:**
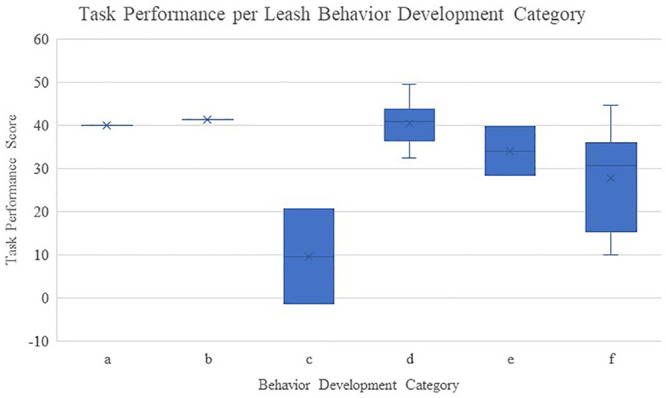
An overview of the task performance of participants per category. For each participant, the average score of the four rounds was calculated. The categorization is based on which category participants were in when looking at their leash activity only: (a) mostly following, (b) start off following, leading in the middle, following at the end, (c) start off following, increase of leading over time, (d) start off leading, increase of following over time, (e) start off leading, following in the middle, leading at the end, (f) mostly leading.

We categorized each dimension of behavior (leash activity, participant movement and participant location) into one of those types of leadership behavior development for each participant. This resulted in a very wide distribution of behavior, showing that participants engaged in highly personal ways of dealing with leadership roles and shifts in the context of the task. While many participants could be categorized in the same type of behavior for at least two of the dimensions (meaning that participants themselves behaved relatively consistently), the pattern of combined dimensions was unique for almost every participant. For a distribution of participants across the behavior development types, see Table 5. To understand how these types of behavior relate to task performance, we created a boxplot of the task performance related to each category of behavior development, using the categorization based on leash activity (Figure 4). Given the small number of participants, it is impossible to draw any hard conclusions from this (especially about category (a) and (b), as only one participant was categorized in either of those). Realistically, only (d) and (f) provide relevant information since both these categories contain 6 participants; it is interesting to see that in this case, the category that is more balanced (d) indeed scores better than the category in which participants were strongly leading all the time (f).

**TABLE 5:** An overview of the distribution of participants across all six behavior development types for each behavior dimension (leash activity, participant location and participant movement).

	**Leash activity**	**Participant location relative to robot**	**Participant Movement**
Mostly following (a)	4 (n = 1)	4, 15 (n = 2)	15, 11 (n = 2)
Start off following, leading in the middle, following at the end (b)	13 (n = 1)	13 (n = 1)	18, 13 (n = 2)
Start off following, increase of leading over time (c)	14, 1 (n = 2)	7, 14, 10 (n = 3)	2, 12, 14, 1, 10 (n = 5)
Start off leading, increase of following over time (d)	3, 16, 7, 18, 15, 11 (n = 6)	3, 18 (n = 2)	8, 6, 3, 16, 7, 4 (n = 6)
Start off leading, following in the middle, leading at the end (e)	5, 12 (n = 2)	5, 12, 16, 1 (n = 4)	5 (n = 1)
Mostly leading (f)	9, 17, 2, 8, 6, 10 (n = 6)	9, 17, 2, 8, 6, 11 (n = 6)	9, 17 (n = 2)

*Each number represents a participant.*

#### Subjective Collaboration Fluency and Leadership Roles

Besides behavioral data, subjective Collaboration Fluency was also measured after each round of performing the task using a questionnaire, based on (Hoffman, 2019). We found that the score on this questionnaire increases significantly over time. This effect was visible within three runs of performing the task. This means that regardless of how people behave, the way in which participants interacted with the robot enabled them to develop a more fluent collaboration over time.

We also found that there was a weak (but significant) negative correlation between the Collaboration Fluency score and the amount of leading behavior people expressed through the leash and movement. This means that when participants were less willing to follow the robot, they also regarded the robot as less cooperative.

### Analyzing Behavior to Uncover Interaction Patterns

The fact that participants were able to develop a fluent collaboration with the robot, while still showing a wide variety of behaviors, prompted us to have a closer look at the specific adaptive behaviors and interactions that emerged in this task. In the following sections, we will explain in more detail how we approached this further analysis as well as the results.

#### Data Analysis: Extracting Interaction Patterns

Using visualizations of the video coding, we studied the videos in more detail, paying specific attention to moments at which adaptations took place (moments at which the codes switched from a leading to a following code for example). We described the specific interactions that we observed at such moments, as well as the interactions of what happened in between those moments. In this process, we tried to focus on the smallest relevant unit of interaction (we will refer to these as unit interactions later in the paper). If it was unclear what a participant was doing at a specific instance, we looked at transcriptions of the interview questions to be able to reliably interpret the intention behind their actions.

The resulting list of interactions was categorized by manually clustering them, after which we described all these different interactions using more general concepts. This process can again be seen as another iteration of open coding: we carefully read each observed interaction and created a code (or sometimes a few codes) to describe the interaction. Within this process we tried to use similar words as much as possible, to keep the list of codes as short as possible. With this process, we aimed to make the interactions less dependent on the specific context of the task executed in the experiment, and more generally applicable. Such more generally applicable interactions are usually called patterns in literature (van Diggelen et al., 2019), and are often used as reference for designing human-technology interactions across different contexts. Important to note here is that patterns are not completely generalizable; they are part of a category of concepts that are called ‘intermediate-level knowledge’(Höök and Löwgren, 2012). They are more abstracted than a single instance, but are not as generalizable as a theory. Their value comes specifically from the fact that they are relatively close to an actual context and task, while being applicable to a range of task and contexts. We will call the more generalized versions of the observed interactions *interaction patterns*. Besides a specification of these interaction patterns themselves, we have tried to combine them into sequences to create larger interaction patterns. Also, we have specified how certain interaction patterns related to others. The combination of the set of interaction patterns (the interaction vocabulary) and the details on how they can be combined and relate to each other will be referred to as the *pattern language*.

## Results: Interaction Patterns

Below, we will describe in detail the outcomes of the analysis of the interactions and interaction patterns. As mentioned above, we will describe exactly what interactions were extracted from the video data, how they were categorized and generalized into interaction patterns and how they can be combined into larger sequences.

### Observed Interactions

By analyzing the videos of the collaboration between the human participants and the robot, a list of 34 types of interactions could be distinguished. They are the unit interactions: the smallest relevant co-adaptive interactions than can be described within the context of the experiment. These interactions were categorized in four types:

• Stable situations (10 interactions): interactions observed *in-between* adaptations, such as the interaction of the human leading and the interaction of the robot leading.
• Sudden adaptations (17 interactions): interactions in which the human and/or robot adapted their leader or follower role, therefore starting a transition from one stable situation to another. The adaptation happens in a single moment, over a short period of time, often in response to an event in the task or partner.
• Gradual adaptations (5 interactions): interactions in which the human and/or robot adapted their leader or follower role, therefore starting a transition from one stable situation to another. The adaptation happens gradually over a longer period of time, often in response to a newly hypothesized or discovered property of the partner’s behavior.
• Active negotiations (2 interactions): interactions in which there was a sequence of several short adaptations that eventually also lead to a transition between stable situations.

The full list of these observed interactions and their categorization can be found in Appendix A, but some examples are the following:

• Stable situations: ‘Human speeds forward dragging the robot along’, or ‘robot is in the lead but human actively runs around robot taking into account the route’.
• Sudden adaptations: ‘Human changes direction, thereby loosening the leash, setting the robot free’, or ‘human pulls the leash and moves to the goal when getting close to the goal’.
• Gradual adaptations: ‘Gradually the robot leads more’.
• Active negotiations: ‘Alternating pulling the robot in a specific direction, waiting for the robot to go, then following the robot’.

The behavior of all participants in the experiment can be described as sequences of these unit interactions, thereby generating larger and higher level interactions.

### Interaction Patterns for Adaptive Leader-Follower Behavior

The above described interactions are specifically related to the experimental task. In order to be able to apply them to other contexts, it is necessary to describe them in more general terms. Therefore, we formulated them into general interaction patterns that can appear in any human-robot collaboration where leader-follower dynamics are relevant. Appendix A shows how the observed interactions were described with interaction patterns. Important to note here is that some of the more complex interactions were described using two or three interaction patterns, while some interaction patterns were also used to describe more than one observed interaction. Table 6 presents a list of the resulting more generalized interaction patterns, including their category and a short description.

**TABLE 6:** The interaction patterns identified from the behavioral data, including a description of what they entail.

**Category**	**Concept**	**Description**
Stable situation	Human following	Human lets the robot do its task
	Human actively on top of things, actively supervising	Human is constantly in touch with the robot
	Active observation by human	Human is actively observing what the robot is doing
	Human leading	Human leads the robot
	Human executing the robot’s task	Human executes the task of the robot
	Proactive following by human	Human actively predicts and observes what the robot will do, following their course of action
	Human dragging the robot along while doing all the work, the robot is a burden	Human ignores the robot as much as possible while focusing on completing the task
	Human focuses on their own task, but leaving time for the robot to catch up	Human executes their own task while leaving space for the robot to follow them in that course of action
Sudden adaptation	Unexpected action by a robot team member	The robot does something the human did not expect, possibly triggering a leadership shift
	Human waiting for the robot to finish their task	The human waits for the robot to finish their task, and decides on a leadership role after that
	Human trying to finish the robot’s task when the robot is done	When the robot has finished their task, the human takes over the task to see if it can be improved upon
	Partner-interfering mistake	The robot makes a mistake that directly and strongly interferes with the human’s course of action
	Human losing contact with the robot due to focus on own task	The human focuses very much on their own task, therefore lose contact with the robot
	Being close to finishing the task	The team is very close to finishing the task, which possibly triggers a leadership shift
	Human actively making up for the robot’s limitations	The human foresees a limitation of the robot will hinder their performance, therefore undertakes action to avoid that
	Task achievement	A task achievement is reached, possibly triggering a leadership shift
	Human urging the robot to be more active, ‘come on’	The robot is relatively passive, causing the human to actively urge the robot to be more active
	Human stops with what they’re doing, waits	The human suddenly stops with what they are doing to wait, after which a new leadership role is chosen
	Repeating previous behavior patterns	The human recognizes a situation similar to an earlier situation, and repeats the behavior previously executed
	Human recognizing the autonomy of the robot	The human recognizes the autonomous capabilities of the robot, possibly triggering a leadership shift
	Quick response to leadership shifts due to continuous connection	Due to continuous contact between the team members, a leadership shift initiated by one team member is quickly and smoothly followed by the other
	Robot becomes active after being inactive	After a period of waiting of being inactive, the robot suddenly becomes active again, possibly triggering a leadership shift
Gradual adaptation	Human gradually letting the robot do more	The human gradually lets the robot do more over time
	Human learning to predict the robot’s behavior	Over time, the human gradually gains insight into the robot’s behavior, thereby enabling them to better predict their behavior
	Human trying to regain control in different ways until eventually taking the lead	Over time, the human attempts to take the lead and regain control in different ways, to eventually find a way to keep taking the lead
Active negotiation	Human executing leading in short intervals	The human takes the lead several times in short intervals, observing what the robot does in the following intervals, to actively search for and negotiate a new stable situation
		

The relatively long list of sudden adaptations contains a diversity of interaction patterns. Some of them are triggers for adaptation (e.g., ‘unexpected action by a team member’), while others are outcomes (e.g., ‘team member stops with what they’re doing, waits’). After a closer look at the list we believe that four components can be distinguished within these sudden adaptations:

• External trigger: an event outside of the partner (e.g., in the task, environment or other partner) triggers an adaptation to a new stable situation;
• Internal trigger: an event inside of the partner (e.g., a specific expectation or change of mind) triggers an adaptation to a new stable situation;
• Outcome: a specific action that is preceded by an internal or external trigger, that will gradually develop into a new stable situation afterward;
• In-between-situation: a specific action that is preceded by an internal or external trigger, that serves as a new trigger for adapting to a new stable situation afterward.

To understand how combinations of these components constitute an interaction pattern, each interaction pattern has been described using the above components in Table 7.

**TABLE 7:** The interaction patterns that fall in the category of sudden adaptations described in more detail.

**Interaction Pattern**	**Type of sudden adaptation**
Unexpected action by a robot team member	External trigger
Human waiting for the robot to finish their task	In-between-situation, preceded by trigger of the other partner working on a specific subtask, succeeded by a new stable situation
Human trying to finish the robot’s task when the robot is done	External trigger and outcome
Partner-interfering mistake	External trigger
Human losing contact with the robot due to focus on own task	Internal trigger and outcome
Being close to finishing the task	External trigger, followed by any outcome
Human actively making up for the robot’s limitations	Internal trigger (expectations) and outcome
Task achievement	External trigger
Human urging the robot to be more active, ‘come on’	Outcome, preceded by trigger of the other being inactive
Human stops with what they’re doing, waits	Outcome, preceded by any trigger
Repeating previous behavior patterns	Outcome, preceded by internal trigger
Human recognizing the autonomy of the robot	In-between-situation, preceded by external trigger (behavior of the other), succeeded by a new stable situation
Quick response to leadership shifts due to continuous connection	In-between-situation, preceded by any trigger, succeeded by a new stable situation
Robot becomes active after being inactive	Outcome and internal trigger

**FIGURE 5:**
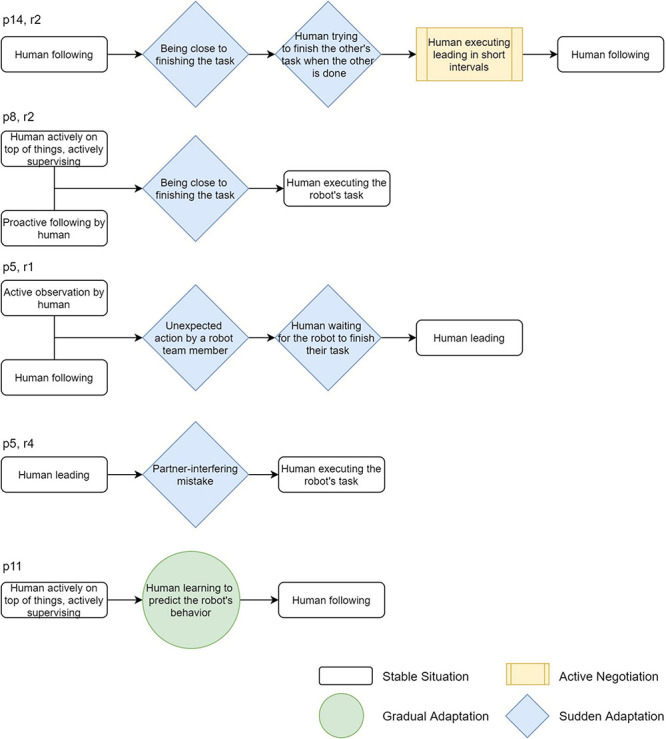
Several example sequences of interaction patterns as they appeared in the experiment.

Using the extended description of the interaction patterns, we can create sequences of them to describe and analyze behavior that participants showed in the experiment. Examples of those are shown in Figure 5. The sequences shown in the figure all represent behavior that participants showed at a specific point in the task. For example, the top sequence is behavior shown by participant 14 in round 2. They were following the robot to pick up the object (stable situation, following). At some point, they were approaching the goal (the robot was also moving toward the goal), which triggered the participant to try to take over the robot’s task by further exploring the field for objects (sudden adaptation, being close to finishing the task and trying to finish the other’s task when the other is done). To urge the robot to follow, the participant pulled the leash in short intervals, but as the robot had already collected all objects, it would continue to move to the goal when the leash was loose (active negotiation, executing leading in short intervals). This resulted in the participant giving in and they again followed the robot (stable situation, following). Another interesting example is the sequence from participant 5, shown in round 4. The participant was focused on reaching the goal (stable situation, leading), when the robot drove over the participant’s feet in an attempt to move with the participant (sudden adaptation, partner-interfering mistake). This caused the participant to immediately take over the robot’s task by exploring the field for objects themselves (stable situation, taking over the other’s task).

From these examples, it can be seen that sometimes different stable situations can exist at the same time to form more complex behavior. Also, different adaptations can happen after each other before a new stable situation is reached. This usually happens when a sudden adaptation is described as an outcome or an in-between-situation. Using sequences of interaction patterns of varying lengths, we can look at the dynamics of co-adaptation at different levels of complexity. This allows us to analyze the effect that small, short-term adaptations have on the overall development of leader-follower roles, but also to dissect large sequences of observed behavior into small units. An explanatory overview of how the observations translate into sequences of interaction patterns is given in a video in Supplementary Material Appendix B.

## Conclusion and Discussion

We have studied the process of co-adaptation within the context of human-robot collaboration. We focused on the adaptations that emerge within the team as a result of interactions around dynamic leadership roles and complementary capabilities. An embodied approach was taken to study subtle and unconscious interactions that manifest themselves in observable physical behavior. We believe that the design of our experiment provides a different way of looking at HRI; one imposes little assumptions about interactions on the design, and that allows for natural interactions based on affordances. In the sections below, we will go into more detail on how the different aspects of our results can be of use for future HRI research and design.

### Interaction Patterns and Team Design Patterns

We have extracted a list of interaction patterns from observed human-robot team behavior. The idea of describing human-robot or human-agent team behavior with patterns has been explored before, such as in van Diggelen et al. (2019); van Diggelen and Johnson (2019); van der Waa et al. (2020), under the name ‘Team Design Patterns.’ In existing research, it is described how these patterns can be useful for designers of human-robot teams, as well as for the actual team members to recognize what activities they are engaged in. These existing *pattern languages* are generally created in a top-down approach. While (van Diggelen et al., 2019) mention that Team Design Patterns can *emerge* from interactions between the human(s) and agent(s) in the team, the pattern languages described in van Diggelen and Johnson (2019); van der Waa et al. (2020) are still designed by the authors of the paper, although the design process is not described in detail. We deliberately use a different name to describe the patterns in our pattern language (interaction patterns instead of team design patterns), because our interaction patterns have not been designed. Rather, they were extracted from existing observations, while they emerged naturally from the context of the human-robot team task. While the Team Design Patterns are very useful, we believe that it is important to also study interactions in human-robot teams in a bottom-up manner, to represent the processes that occur naturally within teams when members collaborate in the real world. The embodied approach of our study enabled us to generate a new interaction pattern language that is based completely on empirical data. It describes the interaction patterns as an emergent feature, while we attempted to keep our own projections of human-only team interactions out of the analysis. Therefore, they can be used as a library of existing natural interactions when designing human-robot interactions; they provide pointers for what natural and fluent co-adaptive HRI can look like.

The approach of studying embodied interactions in a natural setting, and the development of a language to interpret the observed interactions, enabled us to identify the interaction patterns that underly the co-adaptation processes taking place within a team. The interaction patterns can be used in other contexts, other tasks, and other teams, due to our efforts to describe them in a way that is as context-independent as possible. This positions our work as an addition to the work of other researchers (van Diggelen and Johnson, 2019; van der Waa et al., 2020) who study team behavior at a higher level of abstraction, that is more focused on team composition and task division. In the design of and research into human-robot or human-agent teams, both types of pattern languages can be used in different stages of the process. The high-level pattern languages can be used for deciding on team composition and general collaborative interactions, while the elements in the lower level pattern language we describe can serve as pointers for designing the specific detailed interactions between the team members that elicit or support effective team behavior.

### Interaction Pattern Language

The interaction patterns that we have described show that leader-follower dynamics can be described using the concepts of *stable situations, sudden adaptations, gradual adaptations*, and *active negotiations*. They give us a better understanding of the subtleties in leader-follower dynamics: very often it is not so much a matter of leading *or* following, but a bit of both: leadership roles constantly shift, and very often leadership is divided across the team members. The complete pattern language, consisting of interaction patterns as the vocabulary and the connections between them as grammar, provides a framework for analyzing co-adaptive interactions in human-robot collaborations, also in contexts different from the one used in our experiment. Using our pattern language to describe interactions can make it easier to understand why specific role divisions emerge and what can be done to change them.

Moreover, the pattern language can be used by collaborating humans and robots for when they want to communicate about the interactions they are engaged in. The different concepts described by the pattern language can for example be used in a knowledge base for the robot (e.g., in the form of an ontology). This can support the team members in becoming aware of their current leadership roles and possible developments in those roles, to give them more agency in making strategic decisions about the collaboration.

### Relation to Existing Interaction Taxonomies

Our pattern language shows similarities to the interaction taxonomy described in Madan et al. (2015). More specifically, their description of *harmonious interactions* is similar to what we consider *stable situations*, while their description of *conflicting interactions* has overlap with our *sudden adaptations* and *active negotiations*. Our pattern language therefore partly confirms, but also extends their interaction taxonomy. We provide a more detailed description and categorization of their *conflicting interactions*, by expressing the difference between *sudden adaptations* and *active negotiations*, and by also adding *gradual adaptations*. Related to this, we feel that the term *adaptation* is more encompassing than *conflict*, as not all adaptive interactions within these categories come from a directly observable conflict. Moreover, we provide a detailed and task-independent description of the different types of *sudden adaptations*. The extensions originate from the fact that we explicitly focused on interactions that drive co-adaptation, rather than collaborative interaction in general. Moreover, through our extended description of *sudden adaptations*, we provide information on how different interaction patterns relate to each other (i.e. the ‘grammar’ of our pattern language), where in the work of Madan et al. (2015) only the taxonomy is provided (i.e. the ‘vocabulary’). Our interaction patterns are also more detailed than those presented in the existing literature. They are described in such a way that they can also be used to design interactions, rather than to just analyze them.

In terms of the lower level interaction patterns, both the work of Madan et al. (2015) and our work are to some extent related to the task used to obtain them. Their interaction patterns were generated in the context of collaborative object manipulation, while ours were generated within a collaborative navigation context. We, however, explicitly formulated the interaction patterns in such a way that they are generally applicable outside of this initial context. To understand the extent of their generalizability, further evaluation in other task contexts will be useful.

### Limitations

While the list of interaction patterns is quite extensive, it is probably not complete. The specific task context that we used in our experiment of course limits the kind of interactions possible. Also, while the analysis of the data was done in a systematic manner, it is bounded by the frame of reference of the researcher. In order to obtain evidence for the relevance of the proposed language, it is important to attempt to apply the analysis of interaction patterns used here to other tasks. That will provide more insights into the extent of the generalizability of the pattern language, as well as into necessary extensions or adjustments.

Furthermore, there are a few limitations forthcoming from the manner in which the task in the experiment was executed. We claim to study human-robot teamwork, butin our experiment a human operator controlled the robot following pre-configured rules. It may be that the robot behaved different from how a real robot would behave. Moreover, the participants were aware of the fact that the robot was controlled by a human operator, and even though a pilot study showed us that participants did not pay much attention to the operator, it may have still influenced the interactions that emerged. The task was also defined with a relatively low level of agency of the robot, causing the robot to initiate few adaptive behaviors. It is likely that participants noticed this, therefore it might have influenced their initiative to take or delegate leadership. Moreover, we studied a human-robot team in the form of a dyad, whereas the dynamics of team interactions can be very different for other (larger) team compositions. This again stresses the importance of testing the results of the present study in other tasks and contexts and, if possible, with real robots and different team compositions. Outcomes of such studies will help to elaborate and refine the interaction pattern language, eventually enabling a better understanding of co-adaptation in human-robot teams. This, in turn, will support the design of adaptive human-robot teams that are able to operate successfully in the complexity of the real world.

### Final Conclusion

By observing embodied interactions within a human-robot team, we have extracted an interaction pattern language that can be used to describe co-adaptive behavior. This pattern language consists of a list of interaction patterns (the vocabulary) that together make up the different elements of co-adaptation. The interaction patterns can be categorized into stable situations, sudden adaptations, gradual adaptations and active negotiations. Furthermore, the sudden adaptations are built up of external triggers, internal triggers, outcomes and in-between-situations. These categorizations and concepts can be used to link different interaction patterns together, to make sequences of co-adaptive behavior. They can therefore be seen as the grammar of our pattern language.

In future studies, we will use the pattern language to analyze co-adaptive behavior in different tasks and contexts. We will analyze how the presence of certain interaction patterns influences team behavior and performance, to validate how useful the different patterns are in creating successful human-robot teams that make use of fluent co-adaptations.

## Data Availability Statement

The original contributions presented in the study are included in the article/Supplementary Material, further inquiries can be directed to the corresponding author/s.

## Ethics Statement

The study involving human participants was reviewed and approved by the Ethical Review Board of the Industrial Design Department at Eindhoven University of Technology (reference: ERB2019ID7). The participants provided their written informed consent to participate in this study. Written informed consent was obtained from the individual(s) for the publication of any potentially identifiable images or data included in this article.

## Author Contributions

EZ did the main research design, experimentation and analysis, as well as most of the writing. The experiment design and execution was done under supervision of MR, while the data analysis was done under supervision of KB and MN. EB, MR, KB, and MN all reviewed and edited the manuscript at different stages of the process. All authors contributed to the article and approved the submitted version.

## Conflict of Interest

The authors declare that the research was conducted in the absence of any commercial or financial relationships that could be construed as a potential conflict of interest.
